# Spatial learning of female mice: a role of the mineralocorticoid receptor during stress and the estrous cycle

**DOI:** 10.3389/fnbeh.2013.00056

**Published:** 2013-05-30

**Authors:** Judith P. ter Horst, Jiska Kentrop, Marit Arp, Chantal J. Hubens, E. Ron de Kloet, Melly S. Oitzl

**Affiliations:** Division of Medical Pharmacology, Leiden Academic Center for Drug Research and Leiden University Medical Center, Leiden UniversityLeiden, Netherlands

**Keywords:** MR mutant mice, spatial performance, estrous cycle, stress, strategy

## Abstract

Corticosterone facilitates behavioral adaptation to a novel experience in a coordinate manner via mineralocorticoid (MR) and glucocorticoid receptors (GR). Initially, MR mediates corticosterone action on appraisal processes, risk assessment and behavioral flexibility and then, GR activation promotes consolidation of the new information into memory. Here, we studied on the circular holeboard (CHB) the spatial performance of female mice with genetic deletion of MR from the forebrain (MR^CaMKCre^) and their wild type littermates (MR^flox/flox^ mice) over the estrous cycle and in response to an acute stressor. The estrous cycle had no effect on the spatial performance of MR^flox/flox^ mice and neither did the acute stressor. However, the MR^CaMKCre^ mutants needed significantly more time to find the exit and made more hole visit errors than the MR^flox/flox^ mice, especially when in proestrus and estrus. In addition, stressed MR^CaMKCre^ mice in estrus had a shorter exit latency than the control estrus MR^CaMKCre^ mice. About 70% of the female MR^CaMKCre^ and MR^flox/flox^ mice used a hippocampal (spatial, extra maze cues) rather than the caudate nucleus (stimulate-response, S-R, intra-maze cue) strategy and this preference did neither change over the estrous cycle nor after stress. However, stressed MR^CaMKCre^ mice using the S-R strategy needed significantly more time to find the exit hole as compared to the spatial strategy using mice suggesting that the MR could be needed for the stress-induced strategy switch toward a spatial strategy. In conclusion, the results suggest that loss of MR interferes with performance of a spatial task especially when estrogen levels are high suggesting a strong interaction between stress and sex hormones.

## Introduction

Corticosterone modulates learning and memory processes. This action exerted by the hormone is mediated by high affinity mineralocorticoid receptors (MR) and low affinity glucocorticoid receptors (GR) that operate in complementary fashion (Reul and De Kloet, 1985; De Kloet et al., 1998, 2005). The MR is involved in the initial phase of memory formation by appraisal of information, response selection and behavioral flexibility (Oitzl and De Kloet, 1992; Lupien and McEwen, 1997; Berger et al., 2006). Via GR memory consolidation is affected (Oitzl and De Kloet, 1992; Lupien and McEwen, 1997; Oitzl et al., 2010).

Stress is known to affect the performance of both sexes differently in spatial learning tests. In males stress impairs spatial performance (Schwabe et al., 2010a,b) but does not seem to affect or improves spatial learning in females (Bowman et al., 2001; Conrad et al., 2004; Kitraki et al., 2004; ter Horst et al., 2013). As said before the MR is involved in the initial phase of memory formation. Male mice carrying a genetic deletion of MR in the forebrain, the MR^CaMKCre^ mice (Berger et al., 2006), needed more time to learn the spatial circular hole board (CHB) task than their control littermates (MR^flox/flox^ mice) (ter Horst et al., 2012c). During stress the latency to find the exit hole decreased in the MR^flox/flox^ mice, whereas the mutants were not further affected in their performance. A recent study revealed that chronic stress not only affects cognitive performance, but also can change the strategy to learn a task by switching between memory systems by the use of specific brain circuits (Dias-Ferreira et al., 2009). Other studies showed that the switch between memory systems occurs also after exposure to acute stressors. To demonstrate this the CHB can be solved by either using a hippocampus-based “cognitive” spatial strategy or a caudate nucleus-based “habit,” also indicated as a stimulus-response (S-R) strategy (Schwabe et al., 2010a; ter Horst et al., 2012c). After an acute stressor or corticosterone administration, the behavioral strategy can change from spatial to S-R in male rodents (rats: Kim and Baxter, 2001; mice: Schwabe et al., 2008, 2010a; ter Horst et al., 2012c). However, there are profound sex differences. Female mice use both strategies and in response to stress they switch to a spatial strategy, especially when in the estrus phase (ter Horst et al., 2013). The MR is shown to be involved in the choice of the learning strategy (Schwabe et al., 2010a), since the administration of a mineralocorticoid antagonist could prevent in males the stress-induced switch of spatial to habit learning. When stressed male MR^CaMKCre^ mice used the S-R strategy their performance was actually improved as compared to the mutants using the spatial strategy, therefore preventing further deterioration of performance (ter Horst et al., 2012c).

These findings raised the question how genetic MR deletion in the forebrain would affect performance of the female mouse in the CHB over the various stages of the estrous cycle with and without prior exposure to an acute stressor. For instance, during a fear conditioning task female MR^CaMKCre^ mice were unable to extinguish fear memory whereas male mice could (Brinks et al., 2009; ter Horst et al., 2012a), suggesting that loss of MR in the forebrain enhanced sex differences in cognitive and emotional behaviors. To address this question female MR^CaMKCre^ and MR^flox/flox^ mice were subjected to 10 min acute restraint stress 30 min before the training on the CHB started. We report here that, while in the proestrus and estrus stage of the cycle the CHB performance of the control female MR^CaMKCre^ mice was impaired, the exposure to the acute stressor did improve spatial performance, but only of the estrus MR^CaMKCre^ mice.

## Materials and methods

### Animals

Female MR forebrain deficient mice (MR^CaMKCre^; 5–6 months) and female MR forebrain intact littermates (MR^flox/flox^) were bred in the animal facility of Leiden University. The MR^CaMKCre^ mice and their littermates were obtained by breeding MR^flox/flox^ with MR^flox/wtCaMKCre^ mice. A modified MR allele (MR^flox^) was generated in embryonic stem cells of 129Ola mice and the CaMKCre transgene was injected in FVB/N mice (Casanova et al., 2001). The MR^flox^ allele and CaMKCre transgene were backcrossed into C57BL/6J over multiple generations. MR^CaMKCre^ mice have a MR deficiency in the forebrain from postnatal day 12 onwards. For detailed description of the design and breeding of MR^CaMKCre^ mice see (Berger et al., 2006). For this study we have four groups: control MR^CaMKCre^ (*n* = 30), control MR^flox/flox^ (*n* = 25), stress MR^CaMKCre^ (*n* = 18) and stress MR^flox/flox^ (*n* = 21) mice. One week before the behavioral experiment the mice were moved to the experimental room and housed individually in Macrolon cages (translucent plastic: 44 × 22 × 17 cm) with sawdust bedding, a tissue for nest building, tap water and food *ad-libitum*, at 20°C with controlled humidity humidity under a 12 h: 12 h light/dark cycle, lights on at 7.30 h. Experiments were performed between 08.30 and 12.30 h (during the non-active phase of the mice) and were approved by the committee on Animal Health and Care from Leiden University, The Netherlands, in accordance with the EC Council Directive of November 1986 (2010/63/EU).

### Restraint stress

Thirty minutes before the first training trial mice were immobilized for 10 min in a narrow cylinder (transparent Plexiglas; diameter 2.5 cm, 8 cm long) that still allowed breathing but no further movement. Immobilization was performed in a room adjacent to the experimental room. Thereafter, mice returned to their home cage in the experimental room for 20 min.

### Experimental design

#### Apparatus

The CHB is a revolvable round board (gray Plexiglas, 110 cm in diameter, situated 1 m above the floor) with 12 holes at equal distances from each other, 10 cm from the rim of the board. Holes are 5 cm in diameter and can be closed by a lid at a depth of 5 cm. Only when the mouse puts its head over the edge of the hole, the mouse can see whether it is open or not. If open, the hole is the exit to the animal's home cage via a S-shaped tunnel (15 cm long; 5 cm diameter). Numerous cues in the room allow spatial orientation (Schwabe et al., 2010a; ter Horst et al., 2012c). A bottle located close to a hole provides a proximal stimulus, relevant for S-R learning. Total training of the mice on the CHB lasted 1 h and 30 min (6 trials) followed 15 min later by a test trial to reveal the learning strategy.

#### General procedure

***Free exploration trial (FET)***. In the week prior to the FET, mice were trained three times to climb through a tunnel. For the FET mice were placed on the CHB for 5 min. All holes were covered with a lid. A transparent water filled bottle (0.5 L, 22 cm high, 5 cm diameter) was placed next to the hole which is the exit hole during the training trials. At the end of the 5 min, the exit hole was opened and the mouse was gently guided by the experimenter toward the exit hole using an iron grid (20 × 6 cm). This initial exploration trial served to estimate possible differences in mouse exploration behavior. Training trials started one week later.

***Training trials***. Each training trial started by placing the mouse in a gray cylinder (Plexiglas; 25 cm high; 10 cm diameter), located at the center of the CHB. After 5 s, the cylinder was lifted and the animal could explore the board and exit through the tunnel. If a mouse did not enter the exit hole within 120 s, it was gently guided there by the experimenter along the small iron grid. The CHB was cleaned after each trial with 1% acetic acid solution and turned clockwise until another hole was at the location of the exit to avoid an influence of odor cues. The home cage was placed under the exit hole and was not visible to the mouse on the board.

Six successive training trials were given with an inter-trial interval of 15 min. The position of the exit hole was fixed with respect to the distal extra-maze cues in the room. Also, the proximal intra-maze cue (the bottle) was always at the same position next to the exit hole in all six training trials. This set-up allowed that the location of the exit hole could be acquired both by using the relation between spatial cues and by association with a single cue: the bottle.

**Figure 1:**
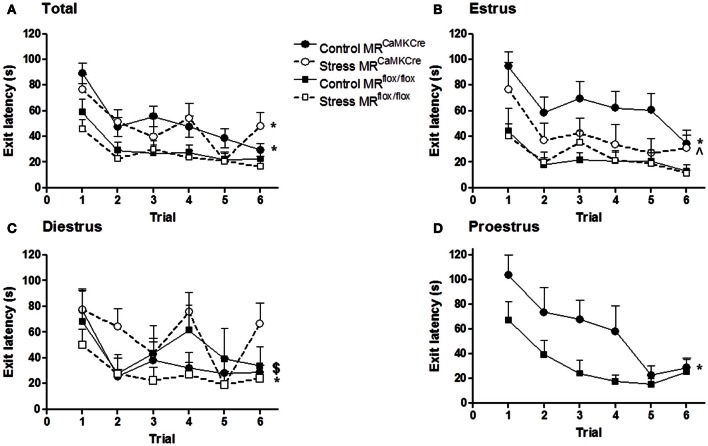
**Estrous cycle and stress affect the latency to exit hole (first visit).** MR^CaMKCre^ (MR forebrain deficient) and MR^flox/flox^ (MR forebrain intact) mice performed six trials on the circular hole board. **(A)** All mice; **(B)** Estrus phase; **(C)** Diestrus phase; **(D)** proestrus phase. Control MR^CaMKCre^ mice (*n* = 30) need more time to find the exit hole compared to MR^flox/flox^ mice (*n* = 25), especially in the proestrus (*n* = 6) and estrus (*n* = 10) phase. Stress has no effect on the latency to exit hole in MR^CaMKCre^ (*n* = 18) and control mice (*n* = 21); however, it improves the performance of estrus MR^CaMKCre^ mice (*n* = 6). Black circles with the solid line are control MR^CaMKCre^ mice and black squares with a solid line represent control MR^flox/flox^ mice. White circles with a dotted line are stressed MR^CaMKCre^ mice and white squares with the dotted line represent stressed MR^flox/flox^ mice. ^*^p < 0.05, compared to MR^CaMKCre^ same estrous cycle phase; ^$^*p* < 0.05, compared to estrus within MR^CaMKCre^ mice; ^∧^*p* < 0.05, compared to control estrus MR^CaMKCre^ mice.

***Revealing the learning strategy***. Trial 7, which started 15 min after the sixth training trial, was a test trial to detect the learning strategy (Figure 1). The exit hole used during the training trials remained open but the bottle was moved to the opposite part of the board and an additional exit hole was opened. Leaving the board through the exit hole used in the training showed the use of a spatial strategy. Using the hole at the novel location and next to the bottle, reflects the use of a S-R strategy. To control for possible odor cues in trial 7, we divided the bedding of the home cage of the mouse over two cages and placed the cages under both exit holes.

### Behavioral observations

Behavior was digitally recorded during the FET and training trials and analyzed with Ethovision XT 6.1 (Noldus Information and Technology BV, Wageningen, The Netherlands). This image analysis system sampled the position of the mouse 12.5 times per second. The following performance parameters were measured for all trials and scored by Ethovision: latency of first visit to exit hole (exit latency: sec), velocity (cm/s), distance moved (cm), latency to leave the board (escape latency: sec) and latency to leave the center (sec; center is 30 cm in diameter). The following parameters were hand scored by the researcher: total number of holes visited (a visit was counted, when the animal put at least its nose in the hole), hole visit errors (the number of holes visited before the exit hole) and perseveration (%). Perseveration was calculated when the mouse visited the same hole twice in a row or when there was one other hole between the two holes (e.g., hole 6, 6 or holes 6, 7, 6). Percentage perseveration is expressed by the number of perseverations divided by the total holes visited.

### Estrous cycle

Smears were taken after the FET and after the training (ter Horst et al., 2013). The mouse was placed on top of its cage, lifted slightly by its tail and the head of the smear loop (size 1 μl; Greiner Bio-one) was gently inserted above the major labia in the cloaca and carefully rubbed along the ventral/rostral side of the cloaca. Cells were transferred to a drop of water on a glass microscope slide, air-dried and stained with Giemsa (Sigma) to facilitate identification of the cycle phase. The four phases are proestrus, estrus, metestrus and diestrus. We did not encounter the metestrus phase which is the shortest and most difficult to detect phase (Caligioni, 2009) so therefore it is not included in this study.

### Statistical analysis

Data are presented as mean ± SEM. Statistical analysis include Chi-square and GLM repeated measures when appropriate. Reported p-values are two-tailed. *P* < 0.05 was accepted as statistically significant. Statistical analyses were performed with SPSS software (version 17.0; SPSS Inc., Chicago, IL).

From two naive MR^CaMKCre^ females the estrous cycle phase could not be determined. The number of stressed MR^CaMKCre^ and MR^flox/flox^ mice in the proestrus phase is too low (*n* = 1–2) to allow test statistics. However, these mice were still included in the “Total” analysis but not used for further sub-analysis.

## Results

Behavior of MR^CaMKCre^ mice differed from the littermate MR^flox/flox^ mice during free exploration and training trials.

### Free exploration trial (FET)

The exploration pattern of MR^CaMKCre^ mice differed from MR^flox/flox^ (Table 1). MR^CaMKCre^ mice were less active than MR^flox/flox^ mice, expressed by slower velocity, shorter walking distance, longer latencies to leave the center and visited less holes (all *p* < 0.05). Furthermore, MR^CaMKCre^ mice persistently returned to holes visited one or two holes earlier (% perseverance, MR^CaMKCre^ mice vs. MR^flox/flox^, *p* < 0.001). These genotype differences in exploration were independent of the estrous cycle. Within MR^CaMKCre^ mice proestrus females showed significantly less perseveration compared to females in other phases [*F*_(2, 33)_ = 4.025, *p* = 0.027; proestrus vs. estrus: *p* = 0.016; proestrus vs. diestrus *p* = 0.01]. The estrous cycle did not affect exploration in the MR^flox/flox^ mice (data not shown).

**Table 1:** **Behavioral data from the free exploration trial (FET)**.

	**MR**^**CaMKCre**^ **(*n* = 48)**	**MR**^**flox/flox**^ **(*n* = 46)**
Velocity (cm/s)	3.57 ± 0.2^***^	4.79 ± 0.2
Distance (m)	10.66 ± 0.5^***^	14.33 ± 0.6
Latency to leave center (s)	13.04 ± 2.7^*^	6.86 ± 1.2
Total holes visited (n)	11.44 ± 0.8^***^	16.07 ± 0.9
Perseveration holes visited (%)	32.01 ± 2.4^***^	18.34 ± 1.9

*** p < 0.0001,

* p < 0.05.

### Training trials

The phase of the estrous cycle had an effect on performance during the training in MR^CaMKCre^ mice but not in their MR intact littermates, the MR^flox/flox^ mice. Statistic data is displayed in Table 2.

**Table 2:** **Repeated measurement (ANOVA) results for behavioral performance parameters during the training on the circular hole board tested for genotype (G), stress (S), and estrous cycle in within controls (ES) effects**.

**Behavioral parameters**		***F***	***df***	***P***
**EXIT LATENCY**				
G	Control	13.103	1.53	0.001^*^
G	Stress	9.450	1.37	0.004^*^
G × S		0.044	1.90	0.833
S	Within MR^CaMKCre^	0.148	1.46	0.702
S	Within MR^flox/flox^	0.622	1.44	0.622
ES	Within MR^CaMKCre^	3.716	2.25	0.039^*^
ES	Within MR^flox/flox^	2.269	2.22	0.127
ES	Control proestrus	10.976	1.15	0.005^*^
ES	Control estrus	22.502	1.19	<0.0001^*^
ES	Control diestrus	0.299	1.13	0.594
ES	Stress estrus	3.805	1.15	0.07
ES	Stress diestrus	6.107	1.17	0.024^*^
**HOLE VISIT ERRORS**				
G	Control	5.740	1.53	0.020^*^
G	Stress	9.275	1.37	0.004^*^
G × S		0.503	1.90	0.480
S	Within MR^CaMKCre^	0.104	1.46	0.748
S	Within MR^flox/flox^	3.785	1.44	0.058
ES	Within MR^CaMKCre^	3.479	2.25	0.046^*^
ES	Within MR^flox/flox^	1.584	2.22	0.228
**LATENCY TO LEAVE CENTER**				
G	Control	16.299	1.53	<0.0001^*^

E.g., control proestrus represents the comparison between control MR^CaMKCre^ and MR^flox/flox^ mice in the proestrus phase

* = significant.

#### Exit latency

Both control genotypes (MR^CaMKCre^ and MR^flox/flox^ littermates) learned the task as demonstrated by the decrease of latencies of first visit of the exit hole over trials [Figure 1A; training: *F*_(5, 265)_ = 12.271, *p* < 0.0001]. However, MR^flox/flox^ mice had significantly shorter latencies than MR^CaMKCre^ mice (Table 2). Taking the phase of the estrous cycle into account showed longer latencies in some estrous phases in MR^CaMKCre^ mice but not in the MR^flox/flox^ mice. MR^CaMKCre^ mice in estrus have significantly longer latencies than diestrus MR^CaMKCre^ mice (*p* = 0.016). Comparing the MR^CaMKCre^ mice to the MR^flox/flox^ mice in different estrous cycle phases shows that proestrus and estrus MR^CaMKCre^ mice have significantly longer latencies to the exit hole than proestrus and estrus MR^flox/flox^ mice; there were no genotype differences in the diestrus phase (Table 2).

After stress both genotypes learned the task as the exit latency was decreasing over trials [Figure 1A; training: *F*_(5, 185)_ = 7.619, *p* < 0.0001]. Stressed MR^CaMKCre^ mice need significantly more time to find the exit hole than stressed MR^flox/flox^ mice. In the stress group we had enough females of both genotypes in the estrus and the diestrus phase but not in the proestrus phase of the estrous cycle. Within the stressed MR^flox/flox^ and the stressed MR^CaMKCre^ mice no estrous cycle effect on the latency to exit hole was found. However, between genotype comparison revealed that stressed diestrus MR^CaMKCre^ mice performed significantly worse on the CHB compared to stressed diestrus MR^flox/flox^ mice whereas a trend was seen in stressed estrus mice (Table 2).

Stress did not affect the performance of both genotypes compared to the controls based on the exit latency (Figure 1). However, stressed estrus MR^CaMKCre^ mice displayed significantly shorter latencies to the exit than control estrus MR^CaMKCre^ mice [*F*_(1, 16)_ = 4.662, *p* = 0.046], whereas stressed diestrus MR^CaMKCre^ had longer latencies than control diestrus MR^CaMKCre^ mice [*F*_(1, 18)_ = 4.304, *p* = 0.053]. Estrus or diestrus stressed MR^flox/flox^ did not behave differently from naive controls in the same estrous phase.

#### Other behavioral parameters

**Figure 2:**
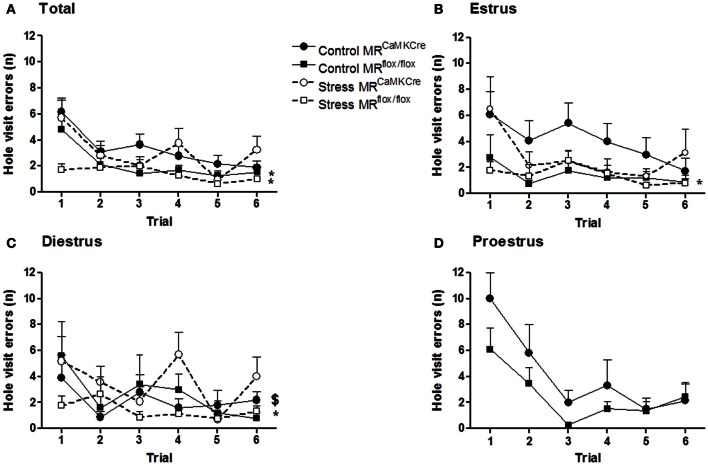
**Hole visit errors in MR forebrain deficient mice during the circular hole board training. (A)** All mice; **(B)** Estrus phase; **(C)** Diestrus phase; **(D)** proestrus phase. Control MR^CaMKCre^ mice make more errors before finding the exit hole compared to MR^flox/flox^ mice, especially in the estrus phase. Stress did not affect hole visit errors, however, stressed MR^CaMKCre^ mice still made more errors than stressed MR^flox/flox^ mice. Black circles with the solid line are control MR^CaMKCre^ mice and black squares with a solid line represent control MR^flox/flox^ mice. White circles with a dotted line are stressed MR^CaMKCre^ mice and white squares with the dotted line represent stressed MR^flox/flox^ mice. ^*^p < 0.05, compared to MR^CaMKCre^ same estrous cycle phase; ^$^*p* < 0.05, compared to estrus within MR^CaMKCre^ mice.

Similar results as in the exit latency are found for hole visit errors (Figure 2). Control and stressed MR^CaMKCre^ mice made more errors than the MR^flox/flox^ mice. An estrous cycle effect in hole visit errors was found in control MR^CaMKCre^ mice but not in control MR^flox/flox^ mice. MR^CaMKCre^ mice in estrus (*p* = 0.025) and proestrus (*p* = 0.049) made significantly more errors than diestrus MR^CaMKCre^ mice. Time to leave the center might also influence the latencies to exit hole. Indeed, MR^CaMKCre^ mice took longer to leave the center than MR^flox/flox^ mice (data not shown). Especially estrus MR^CaMKCre^ mice remained longer in the center compared to proestrus (*p* = 0.021) and diestrus (*p* = 0.003) MR^CaMKCre^ mice. The same significant differences between both genotypes were also found in velocity and time to leave the board (data not shown).

### Test trial

#### Strategy (Figure 3)

To reveal the strategy the intra-maze cue (bottle) was moved to the opposite side of the board. The mice could now exit the board through the original exit hole (spatial strategy) or the new exit hole marked by the bottle (S-R strategy). Independent of genotype control non-stressed mice applied either the spatial (70–80%) or the S-R (20–30%) strategy [χ^2^_(1)_ = 2.509 *p* = 0.285]. No stress effect was seen on strategy use [χ^2^_(1)_ = 0.06 *p* = 0.807] and similar data was seen for the estrous cycle phase.

**Figure 3:**
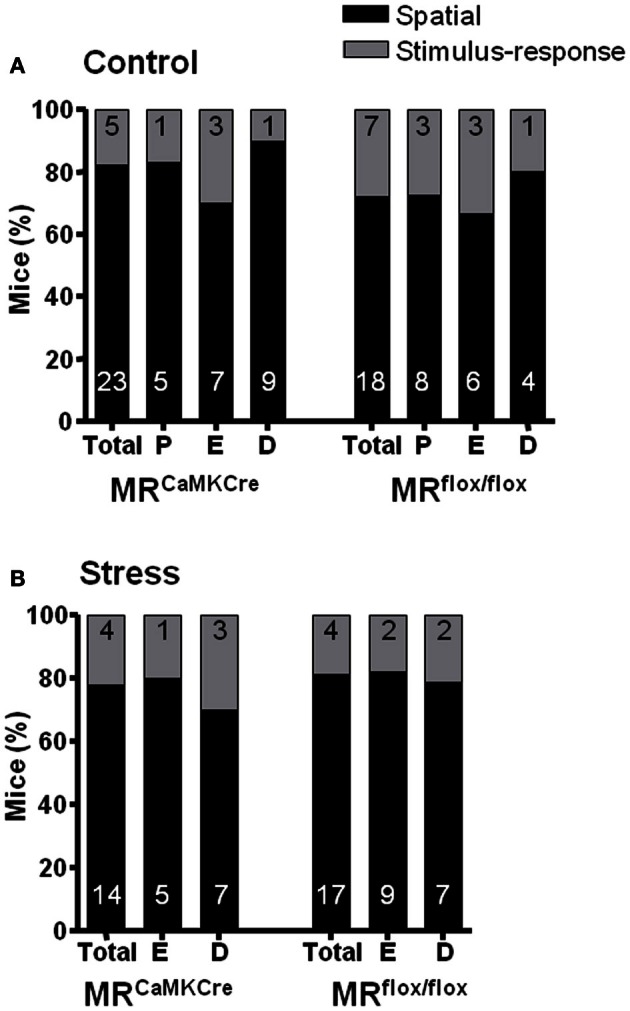
**Percentage of mice using Spatial or Stimulus-response strategies revealed by the test trial.** No effect of the estrous cycle on strategies in **(A)** control and **(B)** stressed mice. Numbers in the bars represent the number of mice using the strategy. Black bar denotes the spatial strategy and the gray bar the stimulus–response strategy. D, diestrus; E, estrus; P, proestrus.

#### Performance

No genotype difference in latency to exit hole was seen during the test trial. Within the control MR^CaMKCre^ mice we found an effect of the estrous cycle [*F*_(2, 25)_ = 3.669 *p* = 0.04; data not shown] with estrus mice taking more time to locate the exit hole compared to proestrus and diestrus mice (40 vs. 10–20 s, *p* = 0.049; *p* = 0.023; respectively). The estrous cycle did not affect the performance of the control MR^flox/flox^ mice during the test trial. Stress did not change the latency to an exit hole in both genotypes.

#### Corrected exit latency

**Figure 4:**
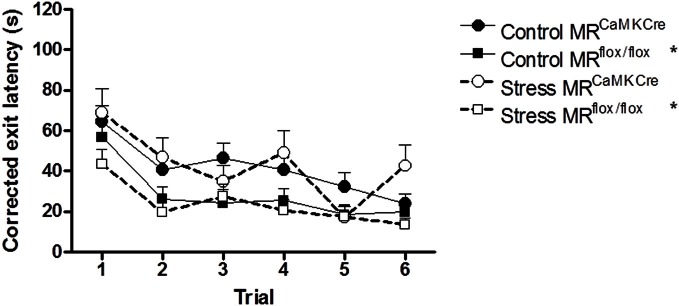
**Corrected exit latency.** Subtraction of the latencies to leave the center from the latency of the first visit of the exit hole still resulted in longer latencies to locate the exit in MR^CaMKCre^ mice compared to MR^flox/flox^ mice, independent of stress. Black circles with the solid line are control MR^CaMKCre^ mice and black squares with a solid line represent control MR^flox/flox^ mice. White circles with a dotted line are stressed MR^CaMKCre^ mice and white squares with the dotted line represent stressed MR^flox/flox^ mice. ^*^p < 0.05, compared to MR^CaMKCre^ mice.

Longer latencies to leave the center could be interpreted as a sign of anxiety interfering with learning. In order to be able to answer the question whether we saw a learning deficit in control and stressed MR^CaMKCre^ or anxiety, the latency to leave the center was subtracted from the latency of first visit of the exit hole. When corrected MR^CaMKCre^ mice still needed significantly more time to find the exit hole compared to the MR^flox/flox^ mice [control: *F*_(1, 53)_ = 6.014 *p* = 0.018; stress: *F*_(1, 37)_ = 8.734 *p* = 0.005; Figure 4).

#### Spatial vs. S-R performance

**Figure 5:**
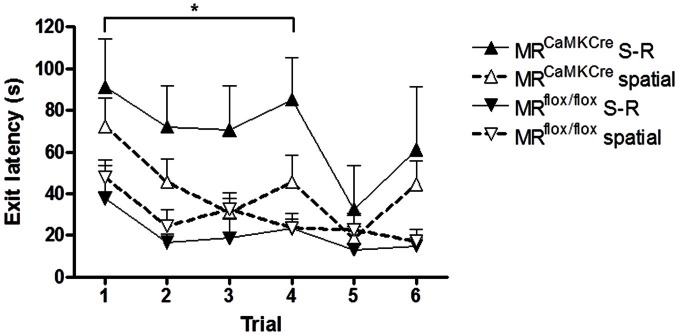
**Exit latency of stressed mice grouped according to the strategy revealed by the test trial.** Stressed MR^CaMKCre^ mice using the stimulus-response strategy (*n* = 4) need more time to find the exit hole compared to MR^CaMKCre^ mice using the spatial strategy (*n* = 14). Strategy (stimulus-response *n* = 4, spatial *n* = 17) did not affect the performance of stressed MR^flox/flox^ mice. Black up-pointing triangles are MR^CaMKCre^ mice and black down-pointing triangles are MR^flox/flox^ mice which used a S-R strategy. White up-pointing triangle represent MR^CaMKCre^ and down-pointing white triangles represent MR^flox/flox^ mice which used a spatial strategy. ^*^p < 0.05, compared to MR^CaMKCre^ S-R strategy users. S-R, stimulus-response.

The performance of mice over the training trials was grouped according to the learning strategy as revealed by the test trial. Stressed MR^CaMKCre^ mice using the S-R strategy performed significantly worse over the first 5 trials compared to MR^CaMKCre^ mice using the spatial strategy [*F*_(1, 16)_ = 4.930 *p* = 0.041; Figure 5) but also compared to stressed MR^flox/flox^ mice using the S-R strategy [*F*_(1, 6)_ = 20.171 *p* = 0.004]. Learning was comparable between stressed MR^CaMKCre^ and MR^flox/flox^ mice using the spatial strategy [*F*_(1, 29)_ = 2.415 *p* = 0.131]. No performance differences between spatial and S-R strategy users were seen in the control MR^CaMKCre^ and MR^flox/flox^ mice and in the stressed MR^flox/flox^ mice [*F*_(1, 28)_ = 1.098 *p* = 0.304; *F*_(1, 23)_ = 0.648 *p* = 0.429; *F*_(1, 19)_ = 0.542 *p* = 0.47; respectively].

## Discussion

In the present study we investigated in female mice the effect of forebrain MR deficiency, estrous cycle and an acute stressor on spatial performance and strategy use by using the CHB. We showed the following findings: Firstly, MR deficiency in the forebrain resulted in longer latencies to the exit hole and more hole visit errors. Secondly, the estrous cycle phases did not affect spatial performance in the wild type mice (MR^flox/flox^) but did in the mutants. The MR^CaMKCre^ mice in the proestrus and the estrus phase had longer latencies to the exit hole compared to MR^CaMKCre^ mice in diestrus. Thirdly, prior stressor exposure did not affect performance on the CHB of the MR^CaMKCre^ or MR^flox/flox^ mice. And finally, stressed estrus MR^CaMKCre^ mice learned the CHB significantly faster than control estrus MR^CaMKCre^ mice.

Around 70% of the control female mice (MR^CaMKCre^ and MR^flox/flox^ mice) favored the spatial strategy over the S-R strategy. Stress and estrous cycle did not influence the strategy use. However, stressed MR^CaMKCre^ mice using the S-R strategy needed significantly more time to find the exit hole compared to spatial strategy users.

### Loss of mineralocorticoid receptor impairs spatial learning

Female MR^CaMKCre^ mice needed significantly more time to find the exit hole on the CHB compared to the MR^flox/flox^ mice. However, detailed analysis showed that the MR^CaMKCre^ mice stayed longer in the center than MR^flox/flox^ mice before moving toward the exit hole. Could it be that they are slower to start their exploration or is this maybe an anxiety response? MR forebrain deficiency caused no differences in anxiety (Berger et al., 2006). Although, MR overexpression in mice resulted in reduced anxiety (Lai et al., 2007; Rozeboom et al., 2007). When the exit latency was corrected for the time spent in the center we still saw a significantly increased latency to the exit hole in the MR^CaMKCre^ females compared to MR^flox/flox^ mice. This implies that the impairment found in the MR^CaMKCre^ mice is mainly due to a slower start of exploration than due to increased anxiety. Male MR^CaMKCre^ mice also needed more time to find the exit hole compared to MR^flox/flox^ littermates on the CHB (ter Horst et al., 2012c). However, male MR^CaMKCre^ mice did not remain longer in the center (ter Horst, unpublished observation). Furthermore, in the Morris water maze MR^CaMKCre^ mice were found to be more passive and needed more time to learn the task (Berger et al., 2006), which is comparable to the behavior seen on the CHB.

Another observation was that the MR^CaMKCre^ mice made more hole visit errors than MR^flox/flox^ mice. MR^CaMKCre^ mice were reported to make also more re-entry errors on the radial arm maze, especially females (Berger et al., 2006). In addition, during the free exploration trial female MR^CaMKCre^ mice were found to return to the same holes repeatedly (perseveration) while the total number of visited holes was comparable to the MR^flox/flox^ mice. To support this finding, male MR^CaMKCre^ mice were also reported to show this intense exploration or hyper-responsiveness (ter Horst et al., 2012c). Moreover, in a novel object recognition task male and female MR^CaMKCre^ mice persistently explored the novel object more than their MR forebrain intact littermates. Furthermore, high emotional arousal was observed in stressed female MR^CaMKCre^ mice compared to MR^flox/flox^ mice (Brinks et al., 2009). Alternatively, MR forebrain overexpressing mice also showed a persistent searching for an absent platform in the water maze suggesting perseveration (Harris et al., 2013).

The present study, therefore, shows that MR forebrain deficiency impairs spatial performance in females most likely due to a change in exploration patterns.

### The phase of the estrous cycle affects the spatial performance of control MR^CaMKCre^

The main question was whether the phase of the estrous cycle could interfere with spatial learning. Here, we saw that control MR^flox/flox^ mice were not affected in their spatial performance by the estrous cycle phases. In concordance, previously the estrous cycle phase did not interfere with the spatial learning in C57BL/6J female mice on the CHB (ter Horst et al., 2013), which is the background strain of the MR^CaMKCre^ mice (Berger et al., 2006). Furthermore, Berry et al. (1997) found no estrous cycle effect on spatial performance. However, the investigators that did find an effect of the estrous cycle phase on spatial learning reported either an impairment in the proestrus (Bowman et al., 2001; Pompili et al., 2010) or in the estrus phase (Frye, 1995; Healy et al., 1999; Frick and Berger-Sweeney, 2001) depending on the task, species and strain.

Sex hormones fluctuate during the estrous cycle. During the proestrus phase estrogen levels are high whereas during the estrus phase a small peak in progesterone is found but the estrogen levels are at their lowest at the end of this phase (Walmer et al., 1992). In these phases spatial learning was impaired in the MR^CaMKCre^ mice. Being in the diestrus phase of the estrous cycle did not affect the spatial performance in the MR^CaMKCre^ mice. During the proestrus synapse formation and dendritic spine numbers in hippocampal CA1 neurons in rats is highest whereas in the estrus it rapidly decreases and intermediate in the diestrus (Woolley et al., 1990a; Shors et al., 2001). Carey et al. (1995) elegantly demonstrated that estradiol decreases hippocampal MR mRNA expression and binding capacity. Progesterone binds to MR, and acts as an antagonist (Carey et al., 1995). In addition, progesterone produces an increase in MR mRNA *in vivo* but only after estrogen pretreatment (Castren et al., 1995). Clearly there is a strong interaction between sex- and stress hormones with regard to MR function. MR forebrain deficiency impairs spatial learning in mice, especially at proestrus (high estrogen levels) and estrus (decreasing estrogen levels with a small peak in progesterone levels).

### Acute stress did not influence spatial performance in female mice independent of the MR

Mice were acutely stressed in this study by a 10 min restraint starting 30 min before the first training trial. Previous studies with male mice showed that corticosterone levels were increased after the acute stress (Schwabe et al., 2010a; ter Horst et al., 2012c). The lack of effect of the acute stress on the learning of the CHB in female mice is supported by data from the background strain, C57BL/6J, which also did not show a stress effect on spatial performance in female mice (ter Horst et al., 2013).

In female mice and rats the effect of stress on spatial learning depends on the type, duration, and timing of the stressor (ter Horst et al., 2012b). Furthermore, pretraining familiarizes the animals to the task and apparatus and reduces novelty stress (Bucci et al., 1995; Warren and Juraska, 1997). Chronic stressed (isolation or restraint) female rats showed impaired spatial performance (Daniel et al., 1999; Conrad et al., 2003). However, chronic restraint stress as well as acute stress could also improve spatial learning (Bowman et al., 2001; Conrad et al., 2004; Kitraki et al., 2004). Stressed female mice by predator odor showed improved learning in the Morris water maze (Galliot et al., 2010) while acute restraint stress did not affect their performance on the CHB (ter Horst et al., 2013).

In contrast to females, both chronic and acute stress impaired spatial performance in male rats (Luine et al., 1994; Diamond et al., 1999; Bowman et al., 2001; Conrad et al., 2003, 2004; Kitraki et al., 2004). Also male mice showed impaired spatial performance on the CHB after acute (Schwabe et al., 2010a) and chronic stress (Schwabe et al., 2008). However, acute stress did not worsen the spatial performance in the male MR^CaMKCre^ mice but did so in their littermates (ter Horst et al., 2012c). A possible explanation for this could be that the MR^CaMKCre^ mice already have increased basal corticosterone levels when compared to MR^flox/flox^ mice (ter Horst et al., 2012c), which was also found in female MR^CaMKCre^ mice (ter Horst et al., 2012a).

Chronic stress produced hippocampal CA3 synapse loss and dendritic retraction in males (Woolley et al., 1990b; McLaughlin et al., 2007). In female rats, chronic stress either failed to influence CA3 dendritic arbors or showed a mild hippocampal CA3 dendritic retraction (Galea et al., 1997; McLaughlin et al., 2010). However, chronic stress combined with estradiol increased the dendritic complexity of the CA1 region in either ovariectomized or gonadally intact females (McLaughlin et al., 2010; Conrad et al., 2012). This suggests that sex hormones might prevent stress induced hippocampal CA3 induced neuronal loss and in parallel impair spatial performance.

### Interaction effect of acute stress and the estrous cycle phase on spatial learning in MR^CaMKCre^

Acute stress did affect the performance of the MR^CaMKCre^ mice on the CHB depending on the estrous cycle phase. This interaction effect of acute stress and the estrous cycle phase was not observed in the littermates. Stressed estrus MR^CaMKCre^ females showed improved performance on the CHB compared to naive MR^CaMKCre^ mice in estrus. The opposite effect was seen in diestrus stressed MR^CaMKCre^ females: decreased performance compared to naive MR^CaMKCre^ mice in diestrus. Thus, in absence of MR, the impact of acute stress is larger with better performance in estrus and worse performance in diestrus. Whether this aggravation of the stress effect is because of altered stress-induced circulating sex steroid levels (Burgess and Handa, 1992; Shors et al., 1999) or because of dysregulation or a shift of the estrous cycle (Pollard et al., 1975; Herzog et al., 2009) remains to be investigated.

### Problem-solving strategy is not affected by sex and stress hormones

To solve the CHB task mice can either use a spatial or a S-R strategy (Schwabe et al., 2010a). The spatial strategy relies on multiple stimuli in the surrounding environment and on the functionality of the hippocampus (White and McDonald, 2002). The S-R strategy makes use of one single proximal stimulus and requires the functionality of the caudate nucleus (Packard and Knowlton, 2002; Schwabe et al., 2010a). Male C57BL/6J mice have a preference for the spatial strategy (Schwabe et al., 2008, 2010a; Bettis and Jacobs, 2009) as do male MR^CaMKCre^ mice (ter Horst et al., 2012c). Here, control MR^flox/flox^ and MR^CaMKCre^ females used either the spatial or the S-R strategy, as did female C57BL/6J mice (Bettis and Jacobs, 2009; ter Horst et al., 2013) and female rats (Tropp and Markus, 2001; Korol et al., 2004; Pleil and Williams, 2010; Hawley et al., 2012). Clearly, there are sex differences in strategy use.

In this study the estrous cycle phase did not affect strategy choice in control, hence non-stressed, female mice, as was seen before on the CHB in C57BL/6J mice (ter Horst et al., 2013). In female rats the phases of the estrous cycle did not affect the strategy on the T-maze (Hawley et al., 2012). However, others reported that female rats in the proestrus phase predominantly used the spatial strategy in a T-maze task (Korol et al., 2004; Pleil and Williams, 2010) and in the estrus phase were strongly affected by the intra-maze cue in a water maze (Sava and Markus, 2005). Cognition and strategy use in female mice and rats depend on multiple factors such as the learning task self (simple or complex) but also in which memory phase is tested (acquisition or memory retrieval).

Stress did not affect the strategy choice in female MR^CaMKCre^ and MR^flox/flox^ mice on the CHB. So far the effect of stress on strategy choice has scarcely been examined in females. In C57Bl/6J female mice a stress-induced switch toward spatial was seen, especially in estrus females, thereby improving their performance on the CHB (ter Horst et al., 2013). That we did not find a stress-induced switch toward a spatial strategy in this study could result from the fact that the majority of the mice (70–80%) already used a spatial strategy, whereas in the previous study 50% of the mice employed a spatial strategy. We do not know why in the present study the mice preferred the spatial strategy. Nevertheless, the switch from S-R toward a spatial strategy after cortisol administration was also found in women (Schwabe et al., 2009). Acute and chronic stress in males make them switch toward a more S-R strategy (Schwabe et al., 2007, 2008, 2010a). Interestingly, in males the MR was found to be involved in the stress-induced switch from spatial to the S-R strategy and this switch rescued their performance (Schwabe et al., 2010a; ter Horst et al., 2012c). Even though we did not see a stress-induced strategy switch in this study, acutely stressed female MR^CaMKCre^ mice using a S-R strategy performed significantly worse on the CHB compared to spatial strategy users. This suggests that in females the MR is needed for the stress-induced switch toward a spatial strategy and this switch might rescue their performance.

## Conclusion

Loss of MR in the forebrain results in an impaired spatial performance particularly in proestrus and estrus. This suggests that the MR is necessary for spatial learning when estrogen and progesterone levels are fluctuating. Stress does not influence the spatial performance of controls but it does improve learning in stressed estrus MR^CaMKCre^ mice. Stress or the estrous cycle do not affect the strategy use of both MR^CaMKCre^ and MR^flox/flox^ mice in this study. However, stressed MR^CaMKCre^ using the S-R strategy were significantly impaired on the CHB suggesting that the MR could be needed for the stress-induced strategy switch toward a spatial strategy.

### Conflict of interest statement

The authors declare that the research was conducted in the absence of any commercial or financial relationships that could be construed as a potential conflict of interest.
